# A focused review on laser- and energy-assisted drug delivery for nail disorders

**DOI:** 10.1007/s10103-024-03992-6

**Published:** 2024-01-19

**Authors:** Hailey Konisky, Raquel Klinger, Lesley Coe, Jose A. Jaller, Joel L. Cohen, Kseniya Kobets

**Affiliations:** 1 Albert Einstein College of Medicine, Montefiore Medical Center, 1300 Morris Park Ave, Bronx, NY 10461 USA; 2 AboutSkin Dermatology and DermSurgery, Greenwood Village, CO USA

**Keywords:** Nail disease, Laser-assisted drug delivery, Nail psoriasis, Onychomycosis

## Abstract

The purpose of this review is to consolidate and summarize laser-assisted drug delivery (LADD) for nail diseases, particularly onychomycosis and psoriasis. A PubMed search was conducted in June 2023 using search terms (1) “laser assisted drug delivery” AND “nail,” (2) “laser” AND “nail,” and (3) “nail disorder” AND “laser treatment.” References of papers were also reviewed, yielding 15 papers for this review. Fractional ablative CO_2_ laser (FACL) and Er:YAG laser can be used for LADD of topical medications such as amorolfine, terbinafine, and tioconazole to treat onychomycosis. A fungal culture should be performed to determine the type of dermatophyte, which will help determine which topical will be most effective. Laser settings varied between studies, but overall LADD tended to be more effective than topical treatments alone. Laser-assisted photodynamic therapy (PDT) was also found to be effective in treating onychomycosis. For psoriatic nails, LADD was used to deliver calcipotriol-betamethasone dipropionate foam, tazarotene, triamcinolone, or methotrexate into the nail. Again, LADD was found to be significantly more effective than topical treatment alone. FACL was the only laser noted for use for LADD in both diseases. Laser-assisted drug delivery for nail disease is a newer approach for onychomycosis and nail psoriasis with several benefits and drawbacks. Dermatologists should discuss the option of LADD with their patients who have recalcitrant onychomycosis or nail psoriasis.

## Introduction

Laser-assisted drug delivery (LADD) has recently become popular in treating cutaneous disorders of the skin, nails, and scalp. This method is used to enhance delivery of topical medications. Nail disorders are often hard to treat, as the keratinization of the nail plate prevents topical medications from penetrating the full depth of the nail to the nail bed [1]. Nail disease goes beyond cosmetic concerns, often having psychosocial implications such as pain, decreasing of quality of life, and limiting functional capacity of the hands/feet. Onychomycosis, a fungal infection of the nail plate and/or nail bed, is the most common nail disorder affecting 5.5% of people worldwide (mostly over age 60) [2]. Psoriatic nails are often an indication of a more severe psoriatic condition, and can cause pain and discomfort to patients. Systemic medications are available for both conditions; however, they cannot be used in all patients and not all patients want systemic therapy [2]. The difficulties with traditional treatment for these conditions make laser-assisted topical drug delivery a desirable treatment option. To our knowledge, these are the only two nail disorders currently being treated with LADD, as they were the only disorders that came up in our search of the literature. This focused review highlights LADD treatment in both onychomycosis and nail psoriasis.

## Methods

A PubMed search was conducted in June 2023 by HK and RK using search terms (1) “laser assisted drug delivery” AND “nail,” (2) “laser” AND “nail,” and (3) “nail disorder” AND “laser treatment.” This search resulted in 858 publications. Papers were excluded if they were not in English, if they were review papers, and if they were out of scope for this review, including studies where lasers were used without an accompanying topical medication. When applied, these exclusion criteria left 15 papers to be included in this review.

## LADD in nail diseases

### LADD in onychomycosis

Onychomycosis is a fungal infection of the nail that is notoriously difficult to treat due to the difficulty penetrating the nail plate and the slow growth rate of the nails. There are five clinical types of onychomycosis: distal lateral subungual, proximal subungual, white superficial, total dystrophic, and endonyx. The infection is most commonly caused by dermatophytes, but can also be from non-dermatophyte molds and yeasts [3]. Beyond cosmetic concerns, onychomycosis can become painful and the infection can spread to the surrounding tissue. It can also have psychosocial implications on the patient’s life and relationships. Traditional treatment consists of either topical antifungals, which are often ineffective (1–2% complete cure, 30–50% mycological cure), or oral antifungals, which are slightly more effective but not always well-tolerated and cannot be prescribed to individuals with certain comorbidities (especially liver disease) or medications [4]. Onychomycosis is most often graded using the Onychomycosis Severity Index (OSI), which measures the area of involvement, the proximity of the affected area to the nail matrix, presence of longitudinal streaking, and nail thickness greater or less than 2 mm due to subungual hyperkeratosis. A score of 1–5 corresponds with mild disease, 6–15 is moderate disease, and 16–35 is severe disease. Ablative lasers create fractional “microscopic treatment zones” (MTZs) in the nail plate, allowing for topical medications to penetrate deeper into the nail bed and work more effectively [5]. Secondly, the heat created by the laser is thought to have a fungicidal effect on its own [6]. The following studies demonstrate the efficacy and tolerability of LADD of topical antifungals for onychomycosis (Table 1).

**Table 1:** Summary of onychomycosis articles with laser settings

PMID [ref]	Prevalent infection	Laser/laser settings	Laser protocol	Treatment	Results
Ranjan, E. et al. 35389015 [7]	-	FACL 110 mJ, 256 spots/ cm^2, 0.5-mm pulse interval, 0.1-ms pulse duration, static mode	45 min topical lidocaine 7% w/w + tetracaine 7% w/w under occlusion on the infected nail and periungual area FACL, three passes to the affected nail and 1 mm of surrounding normal nail	Two groups: 200 mg itraconazole twice daily, 1 week on 3 weeks off for 3 months, vs FACL, *four sessions 4 weeks apart* with twice daily 1% terbinafine, assessment at baseline and 6 months	Both groups saw statistically significant improvement in OSI and clinical appearance by 6 months, with no significant difference between groups
Rajbanshi, B. et al. 32314298 [8]	*Trichophyton rubrum*	FACL 10,060 nm, 1.5 mm apart	5% lidocaine cream for 1 h FACL ablation to the affected area until participants reported extreme heat	Two groups: LADD of topical terbinafine vs. terbinafine alone *Three sessions 1 month apart* 6 months terbinafine	Complete clearance at 6 months was achieved in 3.75% of control subjects and 25% of treatment subjects
Zaki, A. M. et al. 31697010 [9]	Yeast, NDM	FACL power 10–15 W, pulse duration 500 μs, space 700–800 μm, stack 3	-	Three groups: FACL vs. 28% tioconazole vs. combined FACL and 28% tioconazole. *Five treatments 3 weeks apart with assessment at 16 weeks*	The combination treatment was significantly more effective in clinical improvement and in mycological clearance at 17 weeks than either treatment alone
Koren, A. et al. 29265166 [10]	*Trichophyton rubrum*	FACL Energy 150 mJ, density 3%, one pass, small spot size	5% EMLA topical numbing for 1 h prior PDT: 3-h incubation, 630-nm light, 75 J/cm^2^	Two groups: FACL delivery of either 20% AMA with PDT or 5% amorolfine lacquer *Six treatments 3 weeks apart*	Both groups did not show a significant improvement when compared to control. Each treatment group saw a considerable increase in positive fungal cultures from 3 to 9 months after treatment
Sobhy, N. et al. 36228978 [11]	*Candida*	FACL 10,600 nm,energy 1.6 mJ, 0.6-mm density IPL 560–700 nm, fluence 12 J/cm^2^	All patients applied topical urea 20% to nails 12 h prior to treatment PDT 20% methylene blue, 3-min incubation, two passes IPL	Three groups: FACL vs. PDT vs. FACL+PDT *6 bimonthly sessions with follow-up 6 months after treatment*	Combination therapy showed higher improvement and success rates than either therapy alone
Abdallah, M. et al. 32419540 [12]	*Aspergillus, Candida*	FACL 180 mJ, 4000-μs pulse duration, density 0.5 mm IPL 640 nm, 18 J/cm^2, 7-ms pulse duration	Overnight application of 40% topical urea 1 week prior to PDT FACL two passes, 3x overlap to the affected nail	Two groups: FACL+PDT vs. PDT alone *6 bimonthly sessions, with follow-up at weeks 24 and 36*	Both groups saw significant reduction in OSI; however, clinical improvement and patient satisfaction were significantly higher in the combined group
Lim, E. et al. 24655819 [13]	*Trichophyton rubrum*	FACL pulse 160 mJ, density 150 spots/cm^2, static mode	30-min topical anesthesia FACL, two to three passes to affected area and 1 mm surrounding normal nail	One group: FACL plus once daily topical amorolfine *Three treatments 4 weeks apart, assessed at 3 and 6 months*	92% of subjects had a clinical response and 50% had a complete response with negative culture after the final treatment
Bhatta, A. et al. 26874820 [14]	*Trichophyton rubrum*	FACL pulse 99 mJ, density 410 spots/cm^2, pulse interval 0.5 mm, static mode	FACL, two to six passes depending on severity to affected area and 1 mm surrounding normal nail	One group: FACL plus once daily topical amorolfine *Three laser treatments 4 weeks apart, assessed at 3 and 6 months*	92% of subjects were culture negative at 3 months and 80% were culture negative at 6 months
Shi, J. et al. 28557542 [15]	*Trichophyton rubrum*	FACL Deep mode, energy of 15 mJ, pulse duration 0.01–0.99 s, and a density of 15%	FACL, multiple passes to affected area with 2–3-mm margins of normal nail until patient felt mild burning	One group: FACL *12 laser treatments 2 weeks apart* with topical terbinafine, follow-up at months 7 and 9	By month 7 and month 9, there was mycological clearance in 77.42% and 74.19% of nails, respectively. Factors influencing positive outcomes included younger age, nails less than 2-mm thick, first digit nails, and infection with *Trichophyton rubrum*
Abd El-Aal, E. B. et al. 30081698 [16]	*Candida*, NDM	FACL 10,600 nm, 10 W, pulse duration of 500 μs, spacing of 700, stack 3	-	Two groups: both FACL laser *four times 3 weeks apart*, with either 0.1% tazarotene or 28% tioconazole with assessment at 12 weeks	Both groups saw clinical and mycological cures. There was a significant difference between mycological cure rates between groups, which authors postulate may be due to the etiology of the infections
Zhang, J. et al. 27339057 [18]	*Trichophyton rubrum*	Er:YAG laser 2940-nm, fluence 35–62 J/cm^2, density 120 spots/cm^2 per pass, 140 um ablation per pulse minimum	Three passes per session in spiral pattern to entire nail plate. Number of pulse shots depended on thickness of nail and patient pain tolerance	Two groups: 2940-nm Er:YAG laser delivery of 5% amorolfine lacquer vs. 5% amorolfine lacquer monotherapy for 12 weeks *Laser treatment at weeks 1, 2, 3, 4, 8, and 12*	The combination therapy was more effective than monotherapy and significantly reduced the number of nails with positive fungal culture by weeks 12 and 24 (*p*<0.05)
Zhang, J. et al. 32557000 [19]	*Trichophyton rubrum*	Er:YAG laser 2940-nm, fluence of 35–60 J/cm^2, density of 120 spots/cm^2 per pass, 140 um ablation per pulse minimum	Entire nail plate was treated. Number of pulse shots depended on the thickness of the nail and patient pain tolerance	Same as above, with more participants	The combination therapy was more effective than monotherapy in individuals with mild-moderate disease. There was no significant difference in severe onychomycosis

*FACL* fractional ablative CO_2_ laser, *IPL* intense pulsed light, *Er:YAG* erbium yttrium aluminum garnet, *PDT* photodynamic therapy

#### Fractionated CO_2_

Fractional ablative CO_2_ lasers (FACL) create microscopic wounds in the skin that do not damage surrounding tissue, allowing for rapid wound healing. This laser is traditionally used for skin resurfacing to treat photodamage and scarring. It recently gained traction for its ability to facilitate topical drug delivery past the stratum corneum into columns of vaporized nail. Studies have shown that this laser can effectively disrupt the nail plate, allowing for delivery of topical medications deep into the nail bed [5]. A meta-analysis showed that perforated CO_2_ laser was more effective than FACL at treating onychomycosis; however, it was more difficult to control the depth and heat of the laser, which could result in devastating injury to the nail bed [6].

LADD of antifungal medications is a newly studied method of treating fungal nail infections as an alternative to oral medications. Oral itraconazole can cause side effects including but not limited to headache, GI upset, and asymptomatic liver function abnormalities, and is contraindicated in patients with congestive heart failure. One randomized controlled trial compared 4 monthly sessions of FACL with daily 1% terbinafine cream to 200 mg twice daily itraconazole (1 week on and 3 weeks off over 3 months) in 100 participants. Clinical improvement was seen in 84.7% of the laser-treated nails vs 76.5% of the itraconazole group. There was no significant difference in OSI scores between groups at any point during the study. Patient’s experienced fewer side effects and were more compliant with the laser treatment than oral itraconazole, highlighting the use of LADD as an alternative to oral treatment in onychomycosis [7].

An open-label randomized controlled trial compared LADD of topical terbinafine vs. terbinafine alone in 180 participants. FACL-treated participants received treatment once a month for 3 months, and both groups used topical terbinafine under occlusion once daily for 6 months. Complete clearance at 6 months was achieved in 3.75% of control subjects and 25% of treatment subjects (*p*<0.001). The incidence of mycological clearance at 24 weeks was much higher than the control group (OR, 18.27). This study suggests LADD of topical terbinafine is more effective than topical terbinafine alone [8].

One randomized controlled trial compared FACL vs 28% tioconazole vs combined FACL and 28% tioconazole in 120 patients (364 nails). Subjects underwent five laser sessions at 3-week intervals, and tioconazole was used twice daily. They found a significant difference between groups with 55% of combined therapy participants seeing clinical improvements at 5 months versus 30% (laser) and 25% (topical) (*p*<0.005). There was also a significant difference in mycological cure rates following the same pattern, with a negative KOH test in 80% of combined treatment patients, 60% in laser alone, and 55% in topical alone. This study shows that LADD is more effective than either method alone and has the highest patient satisfaction (60% of patients vs 40% vs 30%), correlating with quicker clinical resolution of the infection [9].

An open-label trial of 60 participants with severe bilateral toenail onychomycosis were divided into either group A (laser and 20% aminolevulinic acid PDT vs 20% aminolevulinic acid PDT alone) or group B (laser and topical 5% amorolfine lacquer vs topical 5% amorolfine lacquer alone). Both groups received six treatments at 3-week intervals and were pretreated on one foot with FACL prior to each treatment. While both treatments reduced the number of positive cultures 3 months after the start of treatment (43% positive in LADD+PDT, 50% positive in LADD+ amorolfine), there was no significant difference between combined treatment and the topical treatment control in either group. The authors emphasized the need for studies to include long follow-up periods to ensure lasting efficacy of LADD [10].

A randomized comparative study enrolled 51 participants and split them into three groups: group 1 (six photodynamic therapy (PDT) with methylene blue and IPL), group 2 (six bimonthly FACL sessions), and group 3 (six combined CO2 laser and PDT treatments). Unlike other studies previously mentioned with dermatophyte infections, this study isolated *Candida* from over 60% of its participants. The group with the FACL-assisted PDT saw greater improvement than either therapy alone. By the end of treatment, 35% of group 1, 23% of group 2, and 11% of group 3 still had positive cultures. This study showed that while all treatments showed efficacy at 6 months with no statistical difference among them (*p*=0.253), the combined therapy yielded the highest patient satisfaction (*p*<0.001). The authors postulated that FACL was successful on its own due to removal of infected tissue and direct destruction of the fungus via heat from the laser itself, though the authors caution that the amount of heat from the laser necessary to kill the fungus may not be tolerated by the patient [11]. Another study compared PDT alone vs FACL-assisted PDT in 21 patients. Six sessions of PDT with 2% methylene blue alone or PDT subsequent to FACL were conducted biweekly. At the end of 24 weeks, both methods had significant reduction in positive mycological cultures (76.2% in combined group, 57.1% in PDT only group) and in OSI, though there was no significant difference between groups [12]. These studies demonstrate that both PDT and laser-assisted PDT are effective in treating onychomycosis; however, they suggest that patient satisfaction may be higher in the combined groups due to faster clinical improvement.

One prospective trial used three treatments of FACL delivery of topical amorolfine in 24 participants who failed topical amorolfine treatment alone. At the end of 12 weeks, 92% saw clinical improvement, and 50% saw complete resolution of fungal infection. The authors noted that none of the patients with nails over 2.2-mm thickness saw a complete response to treatment [13]. A similar trial used daily topical 1% terbinafine with laser instead and noted that 92% of subjects were culture negative at 3 months and 80% were culture negative at 6 months [14]. Another trial tested 6 months of topical terbinafine treatment with FACL treatment every 2 weeks in 30 participants (124 nails) with similar results [15].

A randomized trial of 102 participants compared FACL delivery of either topical 0.1% tazarotene or topical 28% tioconazole. Tazarotene, a vitamin A derivative, is thought to have an effect against onychomycosis through both reducing inflammation and keratinocyte proliferation. Both groups received four laser sessions spaced 3 weeks apart. At the end of treatment, there was no significant difference in clinical grading between the two groups; however, more subjects in the tazarotene group had a negative KOH test (91.7% vs. 78.3%, *p*<0.001). The most prominent infection in this cohort was *Candida* followed by non-dermatophyte molds. This study highlights the importance of determining the etiology of nail onychomycosis in order to most effectively utilize LADD for complete remission of the diseased nail [16].

#### Er:YAG

Fractionated erbium yttrium aluminum garnet (Er:YAG) lasers also create columns of “microscopic treatment zones” within nails to more efficiently deliver topical treatments to the nail using a 2940-nm wavelength. A study of four ablative lasers (2940 nm, 2080 nm, 308 nm, and 1053 nm) demonstrated that the 2940-nm Er:YAG laser was most efficient at removing nail tissue [17].

A split hand study was conducted in nine patients with 40 affected nails (both hands had affected nails: one hand treated with 2940-nm Er:YAG laser and 5% amorolfine lacquer and the other hand treated with 5% amorolfine lacquer monotherapy). Laser treatment was conducted in weeks 1, 2, 3, 4, 8, and 12. Both hands received 5% amorolfine lacquer twice weekly. The combination therapy group had significantly decreased Onychomycosis Severity Index scores at both weeks 12 and 24, with the average decreasing by 5.24 points over 24 weeks, whereas the average in the monotherapy group only decreased by 2.09. By 12 weeks, 70% of the combination therapy group and 25% of the monotherapy group had a negative fungal culture [18]. A larger study by the same group found that people with mild-moderate onychomycosis in the combination therapy group showed significant improvement, suggesting that combination 2940-nm Er:YAG laser delivery of 5% amorolfine lacquer is more effective at treating mild-moderate onychomycosis than 5% amorolfine lacquer alone [19]. Both studies demonstrate the efficacy and tolerability of using 2940-nm Er:YAG laser delivery of 5% amorolfine lacquer for onychomycosis [19, 20].

Notably, there are lasers that work synergistically with topical medications to treat onychomycosis rather than physically facilitating drug delivery through microchannels. Neodymium-doped yttrium aluminum garnet (Nd:YAG) lasers use a crystal containing neodymium ions to emit a beam of light at a wavelength of 1064 nm. The Nd:YAG laser beams penetrate the nail plate and heat up the fungal material, causing cellular damage and death of the fungus [21]. Further discussion of these types of and other non-ablative lasers is beyond the scope of this review.

### LADD in psoriatic nails

Nail psoriasis is an autoimmune disease characterized by the rapid proliferation of skin cells in fingernails and toenails, causing onychauxis (nail thickening), pitting of the nail plate, discoloration, and changes in the structure of the nail. Nail psoriasis is commonly associated with a more advanced stage of psoriasis and a higher risk of developing psoriatic arthritis. In addition to medical concerns, nail psoriasis presents cosmetic concerns, pain, and functional impairment. Typical treatment of nail psoriasis includes localized management with topical or intralesional steroids and systemic agents. Unfortunately, topical treatments have not produced successful results due to the inability of the drug to penetrate the nail. Intralesional injection produces unnecessary pain for patients undergoing treatment. LADD shows promise in nail psoriasis treatment, with less pain and greater penetrative ability than traditional treatments. The following studies further discuss the efficacy, safety, and benefits of using LADD in nail psoriasis treatment (Table 2) [22, 23].

**Table 2:** Summary of nail psoriasis articles with laser settings

PMID [reference]	Laser/laser settings	Laser protocol	Treatment	Results
Ortner, V. K. et al. 36321647 [23]	FACL 10,600 nm. Nail plate-10 mJ/mb for 100 μm of OCT-measured thickness. Density 10%	30 min topical anesthetic, pre-cooling. Nail plate treated at baseline, lateral and proximal nail folds treated every time	Two groups: Cal/BD foam treatment with FACL vs. Cal/BS monotherapy *Two treatments 6 weeks apart*	Cal/BD foam is an effective treatment for psoriatic nails. Addition of FACL showed better results, but these results were not significant
Alakad, R. et al. 35333217 [24]	FACL Power 10 W, stack 3, dwell time 500 μs, and spacing 600 μm	-	Two groups: FACL with topical methotrexate vs. intralesional injection of methotrexate *24 treatments 2 weeks apart*	Both groups showed statistically significant improvements in psoriatic signs. FACL with methotrexate had significantly less pain and subungual hematoma than the intralesional group
Nassar, A. et al. 35555966 [25]	FACL Power 10 W, stack 4, dwell time 500 μs, and spacing 550 μm	Topical anesthetic 40 min, one pass to nail plate, proximal, distal, and lateral nail folds	Two groups: FACL with immediate application of triamcinolone acetonide vs. intralesional injection of triamcinolone acetonide *24 treatments 2 weeks apart*	Both treatment groups showed significant improvement in psoriatic signs. Lower pain scores and higher patient satisfaction were seen in the FACL group
Essa Abd Elazim, N. et al. 34664357 [26]	FACL 10,600-nm, pulse energy of 140 mJ, density of 150 spots/cm^2^ and depth level 1	30 min topical anesthetic, two to three FACL passes to affected area depending on depth of nail plus 1 mm of normal nail	Two groups: FACL combined with daily topical tazarotene 0.1% gel vs. daily topical tazarotene 0.1% gel monotherapy *Three treatments 4 weeks apart*	Both treatment groups showed improvements in nail psoriasis, but significantly greater improvement was seen in the FACL and tazarotene 0.1% combination treatment group

*FACL* fractional ablative CO_2_ laser, *Cal/BD* calcipotriene/betamethasone dipropionate foam

Nail psoriasis severity is usually measured using the Nail Psoriasis Severity Index (NAPSI). Nail matrix psoriasis is determined by the presence of any nail pitting, leukonychia, red spots in the lunula, and crumbling. Nail bed psoriasis is measured by the presence of any onycholysis, oil drop, dyschromia, splinter hemorrhages, and hyperkeratosis in any of the four quadrants. A higher NAPSI score corresponds to higher severity of disease [23].

#### Fractionated CO_2_

FACL has also been studied for the treatment of psoriatic nails. Ortner et al. completed a randomized and intrapatient controlled trial of 11 subjects (144 nails) of topical calcipotriol-betamethasone dipropionate foam (Cal/BD) with and without FACL At random, half of the nails from each patient received optical coherence tomography (OCT)-guided 10,600 nm FACL as the treatment group. Even though Cal/BD as monotherapy showed significant improvement in clinical reduction of psoriatic nail activity (N-NAIL -66%, NAPSI -58%), the addition of FACL demonstrated higher efficacy (N-NAIL -85%, NAPSI -78%). However, this difference was not statistically significant, likely due to small sample size. More studies are needed to determine the clinical value of combining FACL with Cal/BD foam treatment [23].

Another study compared LADD of 0.1 mL of topical methotrexate (25 mg/mL) vs intralesional injection of methotrexate (25 mg/mL) in 28 patients (123 nails) with fingernail psoriasis. The control group received intralesional injection of methotrexate (25 mg/mL) and the treatment group received FACL combined with 0.1 mL of topical methotrexate (25 mg/mL). The treatments were given over six sessions at 2-week intervals. Psoriatic signs improved in each group. There were no statistical differences found in NAPSI between the two groups. The FACL group had significantly less pain (*p* < 0.001) and subungual hematoma (*p* = 0.03) than the intralesional group. FACL-assisted delivery of methotrexate shows promising signs as an effective and less painful alternative to intralesional injection of methotrexate. Larger studies are encouraged to confirm the efficacy of FACL-assisted delivery of methotrexate [24].

A similar protocol was used to compare intralesional injection of triamcinolone acetonide (10 mg/mL) and FACL combined with 0.1 mL of topical triamcinolone acetonide (10 mg/mL) in fingernail psoriasis in 34 patients. There was no statistically significant difference found between the two treatments in NAPSI, matrix score, bed score, and dermatoscopic score. The nail matrix and bed psoriatic signs improved significantly in both groups. Higher patient satisfaction (*p* = 0.007) and lower pain scores (*p* = 0.03) were seen in the FACL group. Combination therapy of FACL and corticosteroids may be an effective alternative in the treatment of nail psoriasis [25].

An additional study compared the efficacy of FACL with topical tazarotene 0.1% gel vs topical tazarotene 0.1% gel alone in 32 patients with bilateral fingernail psoriasis. Both hands of each patient received once-daily tazarotene 0.1% gel for 3 months. Additionally, one hand was randomly selected to receive three sessions of 10,600-nm FACL every 4 weeks over this 3-month period. Modified Nail Psoriasis Severity Index (mNAPSI) was measured at 3 and 6 months. Both treatments showed significant improvements in total mNAPSI. However, the combination of FACL and tazarotene showed a higher improvement(48.15%) compared to topical tazarotene alone (31.48%, *p*<0.001). Combination treatment also showed significantly higher patient global assessment scores than topical treatment alone (7.33 ± 2.53 and 5.59 ± 2.41 respectively; *p* < 0.001). FACL with topical tazarotene has been shown to be an effective treatment for nail psoriasis with a significantly better response than topical tazarotene alone. Future studies with longer follow-up periods are needed to determine the chance of disease recurrence after this treatment [26].

There are many benefits to LADD, including deeper penetration of topical medications into the nail plate, which these studies suggest improves the efficacy of the medication. Due to the slow rate of nail growth, patients often give up on their topical treatments before they get a chance to work. Several papers noted quick clinical improvement and high patient satisfaction with LADD treatments, which may inspire patients to adhere to their treatment plans. This results in improved quality of life, mental health, and self-esteem, which are all often negatively impacted by these diseases. Additionally, LADD is an alternative to systemic treatments that have potentially intolerable side effects that may cause hesitation in both the patient and physician, as in many cases these nail diseases are primarily of cosmetic concern. Furthermore, systemic medications like methotrexate, terbinafine, itraconazole, and fluconazole require frequent lab monitoring and are contraindicated in some people, though recent studies suggest this may not be the case for terbinafine in onychomycosis [27]. The limitations of LADD itself include out-of-pocket cost, temporary pain or discomfort during procedure, and the potential unavailability of certain lasers in some dermatology practices. FACL is the only laser shown to be useful in treating both disorders of the nail in the trials we found.

## Conclusion

Laser-assisted drug delivery is a relatively new approach for treating onychomycosis and nail psoriasis. Though the upfront cost of LADD may be higher than topical or systemic treatment, its clinical efficacy, tolerability, and relatively few side effects may offer either an alternative or adjunct therapy option to optimize treatment effect. Larger, more long-term randomized controlled trials are necessary for both diseases to determine the best laser settings and technique. Future onychomycosis trials are highly encouraged to use the gold-standard “complete cure” of onychomycosis as a primary outcome measure rather than clinical improvement or negative culture [28]. Furthermore, these studies highlight the need for a single, standardized, and validated scale for characterizing these nail disorders. We acknowledge that assessing and comparing laser efficacy between studies has limitations due to variation in laser types, laser settings, and clinician technique. Furthermore, it is difficult to assess how much of the nail improvement is due to the formation of microchannels versus the heat of the laser and the ablative destruction of nail pathogens. More research is needed to study how long channels are open in the nail plate and optimal depths of the laser. Overall, LADD is promising and there is an opportunity to expand it to other nail disorders including lichen planus and trachyonychia.

## References

[1] Wenande E, Erlendsson AM, Haedersdal M (2017) Opportunities for laser-assisted drug delivery in the treatment of cutaneous disorders. Semin Cutan Med Surg 36(4):192–201. 10.12788/j.sder.2017.046 29224037 10.12788/j.sder.2017.046

[2] Lee DK, Lipner SR (2022) Optimal diagnosis and management of common nail disorders. Ann Med 54(1):694–712. 10.1080/07853890.2022.2044511 35238267 10.1080/07853890.2022.2044511 PMC8896184

[3] Bodman MA, Krishnamurthy K (2023) Onychomycosis. In: StatPearls [Internet]. Treasure Island (FL): StatPearls Publishing. Available from: https://www.ncbi.nlm.nih.gov/books/NBK441853/

[4] Elewski BE, Ghannoum MA, Mayser P, Gupta AK, Korting HC, Shouey RJ, Baker DR, Rich PA, Ling M, Hugot S, Damaj B, Nyirady J, Thangavelu K, Notter M, Parneix-Spake A, Sigurgeirsson B (2013) Efficacy, safety and tolerability of topical terbinafine nail solution in patients with mild-to-moderate toenail onychomycosis: results from three randomized studies using double-blind vehicle-controlled and open-label active-controlled designs. J Eur Acad Dermatol Venereol 27(3):287–294. 10.1111/j.1468-3083.2011.04373.x 22181693 10.1111/j.1468-3083.2011.04373.x

[5] Tsai MT, Tsai TY, Shen SC, Ng CY, Lee YJ, Lee JD, Yang CH (2016) Evaluation of laser-assisted trans-nail drug delivery with optical coherence tomography. Sensors (Basel, Switzerland) 16(12):2111. 10.3390/s16122111 27973451 10.3390/s16122111 PMC5191091

[6] Ma W, Si C, Kasyanju Carrero LM, Liu HF, Yin XF, Liu J, Xu Y, Zhou B (2019) Laser treatment for onychomycosis: a systematic review and meta-analysis. Medicine 98(48):e17948. 10.1097/MD.0000000000017948 31770202 10.1097/MD.0000000000017948 PMC6890331

[7] Ranjan E, Arora S, Sharma N (2023) Fractional CO2 laser with topical 1% terbinafine cream versus oral itraconazole in the management of onychomycosis: a randomized controlled trial. Indian J Dermatol Venereol Leprol 89(1):47–53. 10.25259/IJDVL_98_2021 dermato-venereologica, 98(4), 467–468. 10.2340/00015555-2874 35389015 10.25259/IJDVL_98_2021

[8] Rajbanshi B, Shen L, Jiang M, Gao Q, Huang X, Ma J, Wang J, Hu Y, Lv H, Wu X, Zhao J (2020) Comparative study of traditional ablative CO2 laser-assisted topical antifungal with only topical antifungal for treating onychomycosis: a multicenter study. Clin Drug Investig 40(6):575–582. 10.1007/s40261-020-00914-6 32314298 10.1007/s40261-020-00914-6

[9] Zaki AM, Abdo HM, Ebadah MA, Ibrahim SM (2020) Fractional CO2 laser plus topical antifungal versus fractional CO2 laser versus topical antifungal in the treatment of onychomycosis. Dermatol Ther 33(1):e13155. 10.1111/dth.13155 31697010 10.1111/dth.13155

[10] Koren A, Salameh F, Sprecher E, Artzi O (2018) Laser-assisted photodynamic therapy or laser-assisted amorolfine lacquer delivery for treatment of toenail onychomycosis: an open-label comparative study. Acta dermato-venereologica 98(4):467–468. 10.2340/00015555-2874 29265166 10.2340/00015555-2874

[11] Sobhy N, Talla Eweed H, Omar SS (2022) Fractional CO2 laser - assisted methylene blue photodynamic therapy is a potential alternative therapy for onychomycosis in the era of antifungal resistance. Photodiagnosis Photodyn Ther 40:103149. 10.1016/j.pdpdt.2022.103149 36228978 10.1016/j.pdpdt.2022.103149

[12] Abdallah M, Abu-Ghali MM, El-Sayed MT, Soltan MY (2022) Fractional CO2-assisted photodynamic therapy improves the clinical outcome and patient’s satisfaction in toenail onychomycosis treatment: an intra-patient comparative single-center study. J Dermatolog Treat 33(1):542–549. 10.1080/09546634.2020.1771252 32419540 10.1080/09546634.2020.1771252

[13] Lim EH, Kim HR, Park YO, Lee Y, Seo YJ, Kim CD, Lee JH, Im M (2014) Toenail onychomycosis treated with a fractional carbon-dioxide laser and topical antifungal cream. J Am Acad Dermatol 70(5):918–923. 10.1016/j.jaad.2014.01.893 24655819 10.1016/j.jaad.2014.01.893

[14] Bhatta AK, Keyal U, Huang X, Zhao JJ (2016) Fractional carbon-dioxide (CO2) laser-assisted topical therapy for the treatment of onychomycosis. J Am Acad Dermatol 74(5):916–923. 10.1016/j.jaad.2015.12.002 26874820 10.1016/j.jaad.2015.12.002

[15] Shi J, Li J, Huang H, Permatasari F, Liu J, Xu Y, Wu D, Zhou BR, Luo D (2017) The efficacy of fractional carbon dioxide (CO2) laser combined with terbinafine hydrochloride 1% cream for the treatment of onychomycosis. J Cosmet Laser Ther: official public Eur Soc Laser Dermatol 19(6):353–359. 10.1080/14764172.2017.1334925 10.1080/14764172.2017.1334925 28557542

[16] Abd El-Aal EB, Abdo HM, Ibrahim SM, Eldestawy MT (2019) Fractional carbon dioxide laser assisted delivery of topical tazarotene versus topical tioconazole in the treatment of onychomycosis. J Dermatolog Treat 30(3):277–282. 10.1080/09546634.2018.1509046 30081698 10.1080/09546634.2018.1509046

[17] Neev J, Nelson JS, Critelli M, McCullough JL, Cheung E, Carrasco WA, Rubenchik AM, Da Silva LB, Perry MD, Stuart BC (1997) Ablation of human nail by pulsed lasers. Lasers Surg Med 21(2):186–192. 10.1002/(sici)1096-9101(1997)21:2<186::aid-lsm10>3.0.co;2-d 9261796 10.1002/(sici)1096-9101(1997)21:2<186::aid-lsm10>3.0.co;2-d

[18] Zhang J, Lu S, Huang H, Li X, Cai W, Ma J, Xi L (2016) Combination therapy for onychomycosis using a fractional 2940-nm Er:YAG laser and 5 % amorolfine lacquer. Lasers Med Sci 31(7):1391–1396. 10.1007/s10103-016-1990-z17 27339057 10.1007/s10103-016-1990-z

[19] Zhang J, Zhang Y, Qin J, Lu S, Cai W, Li J, Huang H, Yang S, Xi L (2021) Comparison of a fractional 2940-nm Er:YAG laser and 5% amorolfine lacquer combination therapy versus a 5% amorolfine lacquer monotherapy for the treatment of onychomycosis: a randomized controlled trial. Lasers Med Sci 36(1):147–152. 10.1007/s10103-020-03054-7 32557000 10.1007/s10103-020-03054-7

[20] Bonhert K, Dorizas A, Sadick NS (2019) Efficacy of combination therapy with efinaconazole 10% solution and 1064 nm Nd:YAG laser for treatment of toenail onychomycosis. J Cosmet Laser Ther: official public Eur Soc Laser Dermatol 21(3):179–183. 10.1080/14764172.2018.1502451 10.1080/14764172.2018.1502451 30052090

[21] Folle L, Fenzl P, Fagni F, Thies M, Christlein V, Meder C, Simon D, Minopoulou I, Sticherling M, Schett G, Maier A, Kleyer A (2023) DeepNAPSI multi-reader nail psoriasis prediction using deep learning. Sci Rep 13(1):5329. 10.1038/s41598-023-32440-8 37005487 10.1038/s41598-023-32440-8 PMC10067940

[22] Canal-García E, Bosch-Amate X, Belinchón I, Puig L (2022) Nail psoriasis. Psoriasis ungueal. Actas Dermosifiliogr 113(5):481–490. 10.1016/j.ad.2022.01.006 35697407 10.1016/j.ad.2022.01.006

[23] Ortner VK, Mandel VD, Skak K, Zibert JR, Bourlioux M, Nissen CV, Fuchs CSK, Philipsen PA, Haedersdal M (2022) Investigating the efficacy and safety of calcipotriol/betamethasone dipropionate foam and laser microporation for psoriatic nail disease-a hybrid trial using a smartphone application, optical coherence tomography, and patient-reported outcome measures. Dermatol Ther 35(12):e15965. 10.1111/dth.15965 36321647 10.1111/dth.15965 PMC10078349

[24] Alakad R, Nassar A, Atef H, Eldeeb F (2022) Fractional CO2 laser-assisted delivery versus intralesional injection of methotrexate in psoriatic nails. Dermatol Surg: official public Am Soc Dermatol Surg 48(5):539–544. 10.1097/DSS.0000000000003418 10.1097/DSS.0000000000003418 35333217

[25] Nassar A, Atef H, Eldeeb F, Alakad R (2022) Comparison of fractional laser-assisted drug delivery and intralesional injection of triamcinolone acetonide in nail psoriasis. Journal der Deutschen Dermatologischen Gesellschaft =. J German Soc Dermatol: JDDG 20(6):788–796. 10.1111/ddg.14731 10.1111/ddg.14731 35555966

[26] Essa Abd Elazim N, Mahmoud Abdelsalam A, Mohamed Awad S (2022) Efficacy of combined fractional carbon dioxide laser and topical tazarotene in nail psoriasis treatment: a randomized intrapatient left-to-right study. J Cosmet Dermatol 21(7):2808–2816. 10.1111/jocd.14536 34664357 10.1111/jocd.14536

[27] Wang Y, Geizhals S, Lipner SR (2021) Retrospective analysis of laboratory abnormalities in patients prescribed terbinafine for onychomycosis. J Am Acad Dermatol 84(2):497–499. 10.1016/j.jaad.2020.04.172 32387655 10.1016/j.jaad.2020.04.172

[28] Yeung K, Ortner VK, Martinussen T, Paasch U, Haedersdal M (2019) Efficacy of laser treatment for onychomycotic nails: a systematic review and meta-analysis of prospective clinical trials. Lasers Med Sci 34(8):1513–1525. 10.1007/s10103-019-02802-8 31254131 10.1007/s10103-019-02802-8

